# The efficacy and safety of haloperidol for the treatment of delirium in critically ill patients: a systematic review and meta-analysis of randomized controlled trials

**DOI:** 10.3389/fmed.2023.1200314

**Published:** 2023-07-27

**Authors:** Jian Huang, Hui Zheng, Xianfeng Zhu, Kai Zhang, Xiaofeng Ping

**Affiliations:** ^1^Department of Critical Care Medicine, Hangzhou Ninth People’s Hospital, Hangzhou, China; ^2^Department of Emergency Medicine, Hangzhou Ninth People’s Hospital, Hangzhou, China; ^3^Department of Critical Care Medicine, Second Affiliated Hospital, Zhejiang University School of Medicine, Hangzhou, China

**Keywords:** haloperidol, delirium, critically ill adult patients, ICU, meta-analysis

## Abstract

**Purpose:**

Delirium is common during critical illness and is associated with poor outcomes. Therefore, we conducted this meta-analysis to investigate the efficacy and safety of haloperidol for the treatment of delirium in critically ill patients.

**Methods:**

Randomized controlled trials enrolling critically ill adult patients to compare haloperidol with placebo were searched from inception through to February 20th, 2023. The primary outcome were delirium-free days and overall mortality, secondary outcomes were length of intensive care unit stay, length of hospital stay, and adverse events.

**Results:**

Nine trials were included in our meta-analysis, with a total of 3,916 critically ill patients. Overall, the pooled analyses showed no significant difference between critically ill patients treated with haloperidol and placebo for the delirium-free days (MD −0.01, 95%CI −0.36 to 0.34, *p* = 0.95, *I*^2^ = 30%), overall mortality (OR 0.89, 95%CI 0.76 to 1.04, *p* = 0.14, *I*^2^ = 0%), length of intensive care unit stay (MD −0.06, 95%CI −0.16 to 0.03, *p* = 0.19, *I*^2^ = 0%), length of hospital stay (MD −0.06, 95%CI −0.61 to 0.49, *p* = 0.83, *I*^2^ = 0%), and adverse events (OR 0.90, 95%CI 0.60 to 1.37, *p* = 0.63, *I*^2^ = 0%).

**Conclusion:**

Among critically ill patients, the use of haloperidol as compared to placebo has no significant effect on delirium-free days, overall mortality, length of intensive care unit and/or hospital stay. Moreover, the use of haloperidol did not increase the risk of adverse events.

## Introduction

Delirium, an acute disturbance in attention and awareness, is a common condition affecting about a third of critically ill patients (1, 2). It is a powerful predictor of prolonged mechanical ventilation, extended length of intensive care unit (ICU) and hospital stay, elevated short-term mortality and worse long-term outcomes (2–5). Notably, critically ill patients treated in ICU have a lot of risk factors associated with ICU therapeutic interventions, including receiving MV, inappropriate sedation and physical restraint (6–8).

The present guidelines advised the multicomponent, non-pharmacological interventions for treatment and prevention of delirium in critically ill patients, including early mobilization, avoidance of oversedation and excess benzodiazepines, family participation, reorientation, cognitive and sensory stimulation (1, 9). Previous studies suggested that these therapy strategies were feasible and safe, had an important role in both treatment and prevention of delirium (10, 11). However, the pharmacologic management of delirium in the ICU remains a subject of debate (12). Current clinical guidelines did not advocate for any particular pharmacotherapeutic intervention in the management of delirium (13, 14). Haloperidol, a highly effective antipsychotic compound, is still the most common treatment for delirium in ICU. An international cohort study investigated 1,260 patients from 13 countries, showed that nearly half of the patients with delirium received haloperidol during the ICU stay (15). Although the clinical benefits of haloperidol for the management of delirium have been proved in non-critically ill patients (14), the use of haloperidol is not supported by existing guidelines because clinical evidence of its effect is limited (1, 16). Furthermore, it has not been approved by the US Food and Drug Administration for the management of delirium as well.

Recently, Andersen-Ranberg and coworkers completed the latest randomized controlled trials (RCTs) to investigate the effect of haloperidol for the treatment of critically ill patients with delirium in ICU (17). The findings suggest that the patients treated with haloperidol did not have a longer survival time at 90 days, as well as the delirium-free and ventilation-free days. To date, both RCTs and meta-analyses have not resolved whether use of haloperidol in critically ill patients had clearly beneficial effects on delirium outcomes. Therefore, we tend to accomplish this updated mate-analysis to further evaluate the effect of haloperidol for the treatment of delirium in critically ill patients.

## Methods

This meta-analysis was conducted in strict accordance with the updated PRISMA statement (18) (Supplementary material 1). The study protocol was preregistered on Open Science Framework.^1^ To identify relevant RCTs meeting our eligibility criteria, we conducted a comprehensive literature search of PubMed, Embase, Scopus, and Cochrane Library from inception up to February 20th, 2023. The literature search was conducted with keywords containing “haloperidol,” “delirium,” “critically ill,” “ICU,” and “randomized.” The full search strategies are given in Supplementary material 2.

### Eligibility criteria

Studies fulfilled the inclusion criteria were included:

1. Type of study: randomized trials;
2. Population: critically ill adult patients (at least 18 years old). If population was unspecified, we deemed the patient population met one of the following criteria to be critically ill patients: the patients enrolled and study concluded in any types of ICU; the patients received therapies which is normally delivered in ICU (e.g., invasive mechanical ventilation); the patients’ illness required intensive care; the patients had been transferred into ICU during study period;
3. Intervention: the use of haloperidol through all routes of administration, without dose limits;
4. Comparison: the use of placebo, or no any type of intervention;
5. Outcomes: the primary outcome of interest were delirium-free days (delirium was assessed by researchers or clinicians from included trials) and overall mortality (including hospital, ICU, 28 day mortality or other. If several mortality rates were reported in one study, we used the mortality at hospital charge in our analysis). Secondary outcomes were length of intensive care unit stay, length of hospital stay, and adverse events.

### Data extraction and quality assessment

Relevant studies were retrieved and their characteristics (including author, years of publication, study design, sample size, characteristics of population, intervention duration and dose, delirium assessment and incidence rate) were extracted by two authors (JH and HZ) independently.

The methodological quality of including studies was independently conducted by two authors (JH and XZ), utilizing the Cochrane risk of bias tool (19). Any discrepancies in the evaluations were resolved through a consensus-based approach, involving a third adjudicator (XP).

### Statistical synthesis and analysis

The odds ratios (OR) with 95% confidence intervals (CI) were calculated using the Mantel–Haenszel method for dichotomous outcomes, and mean difference (MD) with 95% CI were calculated using the inverse variance method for continuous outcomes. The heterogeneity between studies was assessed by the Higgins inconsistency (*I*^2^) statistics (20), substantial heterogeneity was identified when *I*^2^ value > 30%. If no significant heterogeneity existed, we adopted a fixed-effects model to perform the analysis, otherwise a random-effects model was used. In addition, publication bias was evaluated through the use of the funnel plot and Egger’s regression test (21).

To identify potential sources of heterogeneity, a predefined subgroup analysis stratified by population (patients with delirium or without delirium). Furthermore, a sensitivity analysis was performed through the consecutive exclusion of each study to investigate the effect of individual studies.

## Results

### Study identification and characteristics

**Figure 1:**
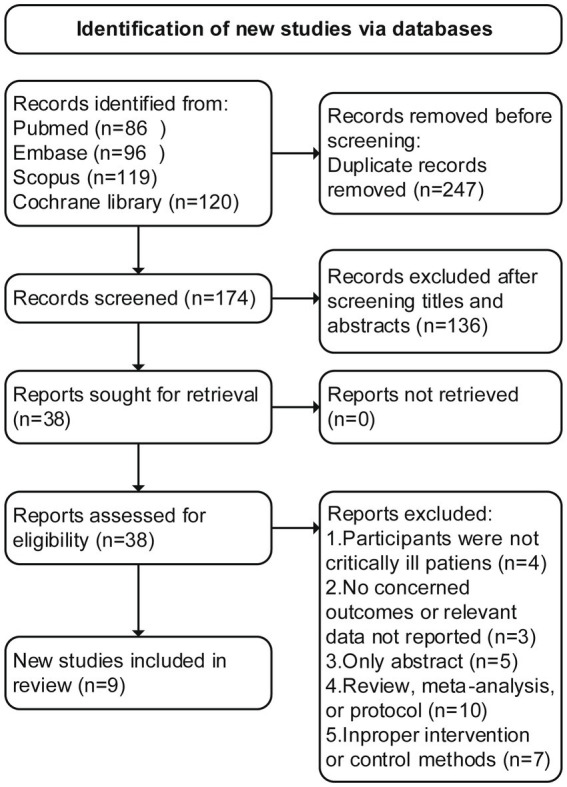
PRISMA 2020 flow diagram for the meta-analysis.

An initial search of the literature resulted in the identification of 421 articles, of which 247 were deemed duplicates and excluded. Through the screening of abstracts, an additional 136 studies were excluded. Following a thorough evaluation of the full text, 29 additional studies were excluded for various reasons (Supplementary material 3 recorded the list of excluded studies). Ultimately, nine RCTs (17, 22–29) met the inclusion criteria and were included in this study, The literature screening flowchart is shown in Figure 1.

The characteristics of the included studies are presented in Table 1. A total of 3,916 patients were analyzed, with 1980 patients receiving haloperidol and 1936 patients receiving placebo during the respective study periods. Different screening tools were used to evaluate the incidence of delirium, including Confusion Assessment Method for Intensive Care Unit (CAM-ICU) (30), Intensive Care Delirium Screening Checklist (ICDSC) (31), Diagnostic and Statistical Manual of Mental Disorders (DSM) criteria. The number of patients in each study ranged from a minimum of 68 up to 1,439. The sample size of six studies (22–27) were relatively small (<400), and the rest of three studies (17, 28, 29) enrolled more than 400 patients. The included studies varied in study population: six trials (17, 22–24, 26, 28) included all critically ill patients, two (25, 29) included patients who were admitted to the surgical ICU postoperatively, and one (27) included elderly patients having emergency admission and high risk for delirium. The incidence of delirium ranged from 16.9 to 100%. In three trials (17, 23, 24), all patients developed delirium during the study period. Van den Boogaard et al. (28) and Wang et al. (29) reported the mortality rate for patients with and without delirium, separately. Different doses, timing and route of administration were also identified: the daily doses of haloperidol ranged from 1.5 to 20 mg, the haloperidol was administered through enteral route in two trials (27, 29) and parenteral route in seven trials (17, 22–26, 28).

**Table 1:** Characteristics of included studies.

Study and year	Design	Number (haloperidol/placebo)	Population	Characteristics (haloperidol/placebo)	Intervention duration and dose	Delirium assessment and incidence of delirium during study period	Outcomes
Andersen-Ranberg (2022)	Multicenter, double-blinded	501/486	Patients ≥18 years old, admitted to ICU and had received a positive result on a screening test for delirium	Age: 70/71; Male (%): 64.7/66.9; Surgical patients (%): 36.5/31.5; Ventilatory support (%): 63.9/62.8	2.5 mg of intravenous haloperidol three times daily until discharge or death in the ICU, up to a maximum of 90 days	CAM-ICU or ICDSC, 100%	90 day mortality, length of hospital stay, delirium-free days, adverse events
Schrijver (2018)	Multicenter, double-blinded	118/124	Patients ≥70 years old, acutely hospitalized through the emergency department for a medical or surgical specialty and at risk for delirium	Age: 83.5/83.4; Male (%): 48.3/41.1; Surgical patients (%): 25.4/21.0; Ventilatory support (%): 63.9/62.9	1 mg of haloperidol through enteral way every 12 h for 7 days	DSM-IV criteria, 16.9%	90 day mortality, length of hospital stay, adverse events
Girard (2018)	Multicenter, double-blinded	192/184	Patients ≥18 years old admitted to ICU with mechanical ventilation, vasopressors, or intra-aortic balloon pump	Age: 61/59; Male (%): 56.3/58.1; Surgical patients (%): 26.6/28.2; Ventilatory support (%): 92.7/92.4	2.5 mg of intravenous haloperidol twice a day for 14-day study period or ICU discharge	CAM-ICU, 100%	90 day mortality, length of ICU stay, length of hospital stay, delirium-free days
Khan (2018)	Single-center, double-blinded	68/67	Patients ≥18 years old received thoracic surgery and admitted to surgical ICU	Age: 60.0/62.3; Male (%): 67.6/71.6; Surgical patients (%): 100/100; Ventilatory support (%): 100/100	0.5 mg of intravenous haloperidol three times daily for a total of 5.5 mg	CAM-ICU, 25.2%	In-hospital mortality, length of ICU stay, length of hospital stay, adverse events
van den Boogaard (2018)	Multicenter, double-blinded	732/707	Non-neurological ICU patients, aged ≥18 years old, with an expected stay >1 day on the ICU	Age: 66.7/67.0; Male (%): 62.7/61.4; Surgical patients (%): 46.0/46.4; Ventilatory support (%): 68.0/64.6	2 mg of intravenous haloperidol three times daily for 28 day study period or ICU discharge	CAM-ICU, 33.1%	90 day mortality, length of ICU stay, length of hospital stay, delirium-free days, adverse events
Al-Qadheeb (2016)	Multicenter, double-blinded	34/34	Mechanically ventilated patients admitted ICU and expected to have an ICU admission at least 24 h	Age: 61.7/59.3; Male (%): 52.9/58.8; Surgical patients (%): 32.4/26.5; Ventilatory support (%): 100/100	1 mg of intravenous haloperidol four times daily for 10 day study period or delirium occurred, or ICU discharge	SAS or ICDSC, 29.4%	In-hospital mortality, length of ICU stay, adverse events
Page (2013)	Single-center, double-blinded	71/70	Patients ≥18 years old admitted to ICU with mechanical ventilation	Age: 67.9/68.7; Male (%): 52.1/64.3; Surgical patients (%): 40.8/30.0; Ventilatory support (%): 100/100	2.5 mg of intravenous haloperidol three times daily for 14 day study period or ICU discharge	CAM-ICU, 67.4%	28 day mortality, length of ICU stay, length of hospital stay, delirium-free days
Wang (2012)	Multicenter, double-blinded	229/228	Patients ≥65 years old who were admitted to ICU after noncardiac surgery	Age: 74.0/74.4; Male (%): 63.3/62.7; Surgical patients (%): 100/100; Ventilatory support (%): 100/100	0.5 mg of intravenous haloperidol bolus injection followed by continuous infusion at a rate of 0.1 mg/h for 12 h	CAM-ICU, 19.3%	28 day mortality, length of ICU stay, length of hospital stay, delirium-free days
Girard (2010)	Multicenter, double-blinded	35/36	Patients ≥18 years old received mechanical ventilation in medical or surgical ICU patients who had an abnormal level of consciousness or receiving sedative or analgesic medications	Age: 51/56; Male (%): 57.1/66.7; Surgical patients (%): 22.9/22.2; Ventilatory support (%): 100/100	5 mg of haloperidol through enteral way every 6 h for 14 days	CAM-ICU, 100%	21 day mortality, length of ICU stay, delirium-free days

CAM-ICU, confusion assessment method for the intensive care unit; ICDSC, intensive care delirium screening checklist; DSM-IV, diagnostic and statistical manual of mental disorders, 4th edition; ICU, intensive care unit.

### Quality assessment

**Figure 2:**
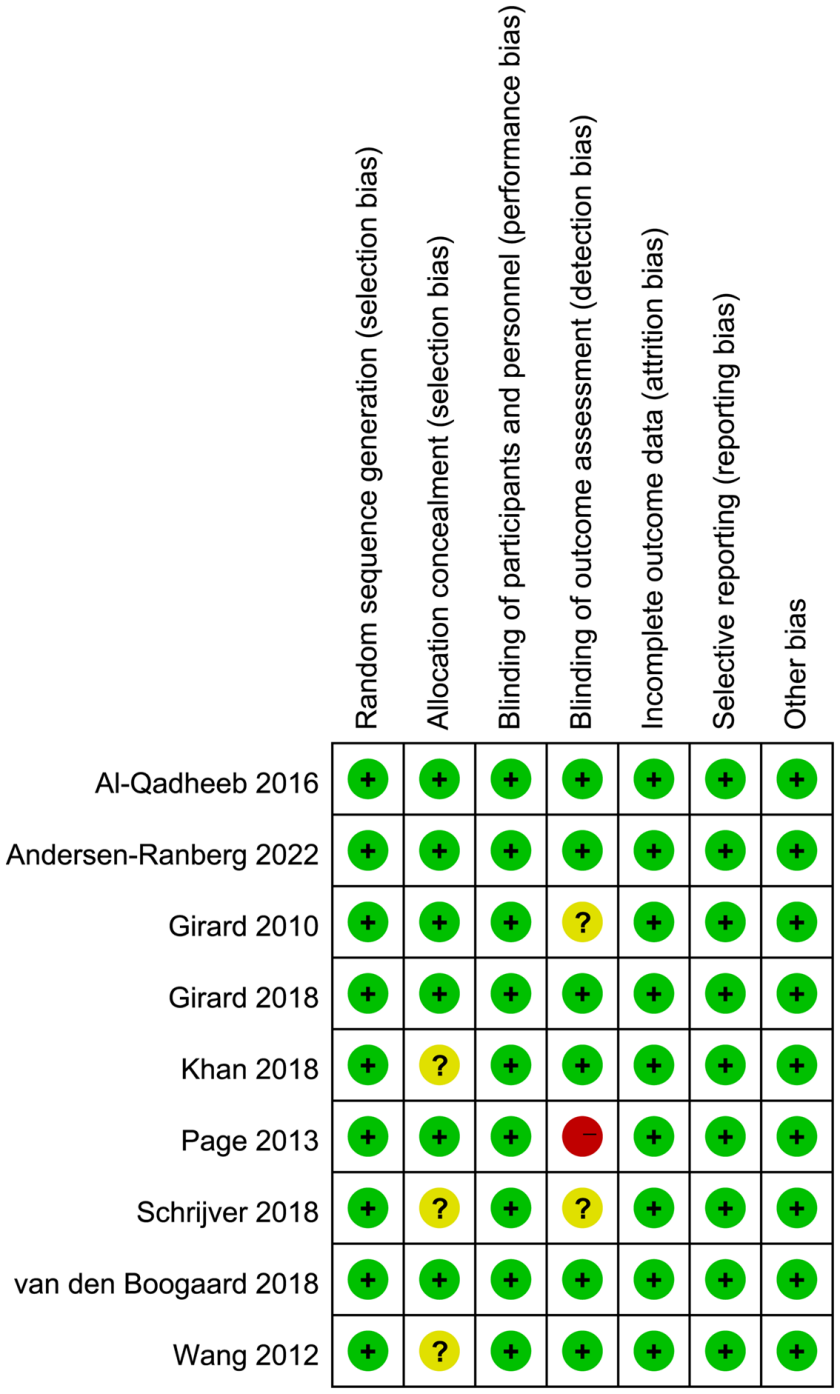
Assessment of quality by the Cochrane risk of bias tool.

The quality assessment of the included studies was conducted using the Cochrane risk of bias tool, and the results are presented in Figure 2. Three studies did not report the details of allocation concealment. For the blinding method for outcome assessment, one trial had high risk of bias since the statisticians were aware of group assignments and treatment allocation, and three trials had unclear risk of bias, which may result in an underestimation or overestimation of the true effect.

We conducted an assessment of publication bias utilizing the Egger’s test and funnel plot, and the results did not indicate a significant risk of publication bias (Egger’s test, *p* > 0.05; Supplementary material 4).

### Primary outcome

**Figure 3:**
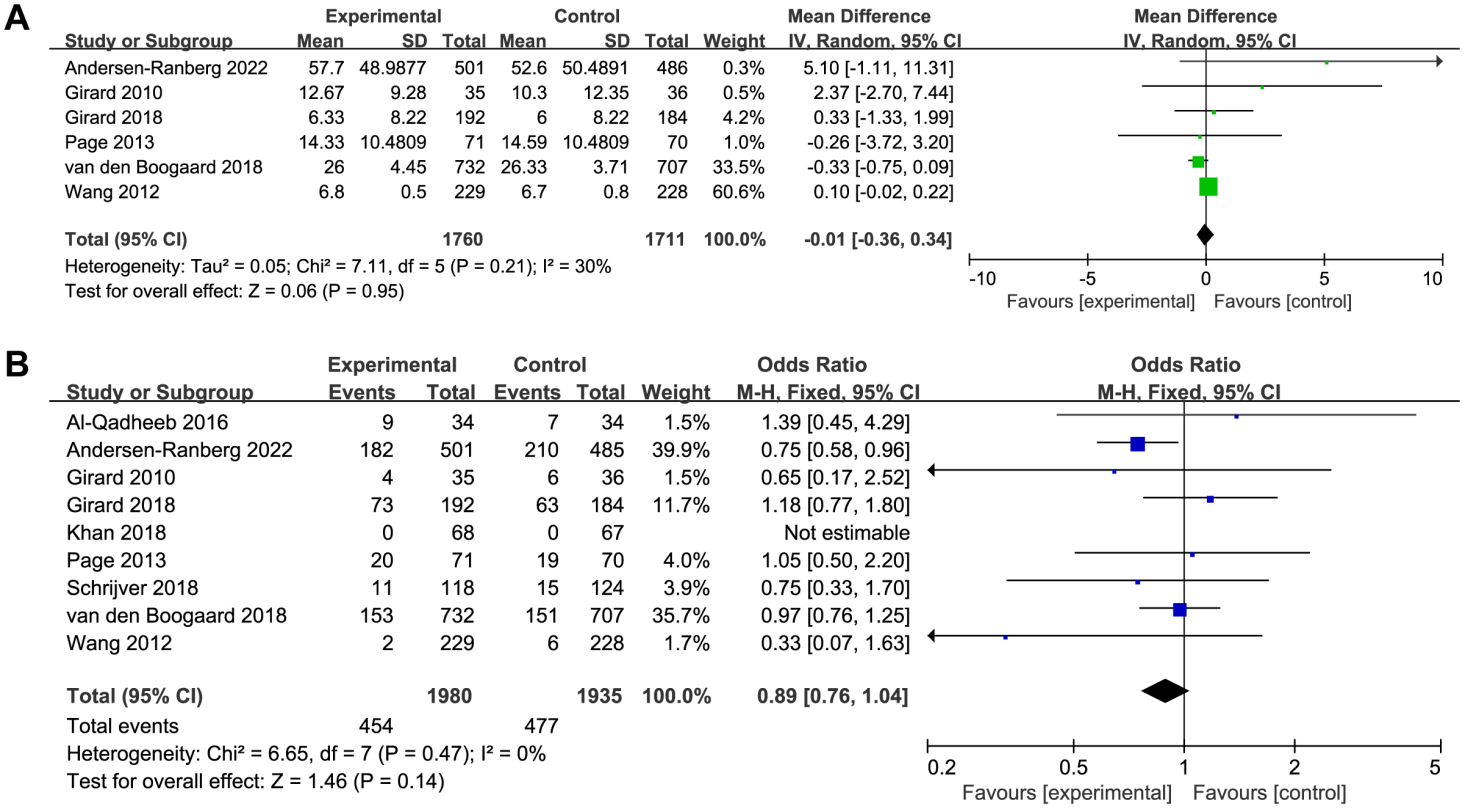
Forest plot showing the association between haloperidol and **(A)** delirium-free days and **(B)** overall mortality.

Six trials reported the delirium-free days and nine trials reported the overall mortality. The delirium-free days was similar between haloperidol and control groups (MD −0.01, 95%CI −0.36 to 0.34, *p* = 0.95, *I*^2^ = 30%; Table 2 and Figure 3A). Similarly, there was no significant difference in overall mortality (OR 0.89, 95%CI 0.76 to 1.04, *p* = 0.14, *I*^2^ = 0%; Table 2 and Figure 3B) between patients received haloperidol and placebo.

**Table 2:** Outcomes of this meta-analysis.

Outcome	*N*	Result
Delirium-free days	6	MD −0.01, 95%CI −0.36 to 0.34, *p* = 0.95, *I*^2^ = 30%
Overall mortality	9	OR 0.89, 95%CI 0.76 to 1.04, *p* = 0.14, *I*^2^ = 0%
Delirium	5	OR 0.85, 95%CI 0.70 to 1.03, *p* = 0.09, *I*^2^ = 19%
Non-delirium	2	OR 0.99, 95%CI 0.72 to 1.35, *p* = 0.93, *I*^2^ = 0%
Length of ICU stay	7	MD −0.06, 95%CI −0.16 to 0.03, *p* = 0.19, *I*^2^ = 0%
Length of hospital stay	7	MD −0.06, 95%CI −0.61 to 0.49, *p* = 0.83, *I*^2^ = 0%
Adverse events	8	OR 0.90, 95%CI 0.60 to 1.37, *p* = 0.63, *I*^2^ = 0%

*N*, number of studies; ICU, intensive care unit; OR, odds ratio; MD, mean difference; CI, confidence interval.

**Figure 4:**
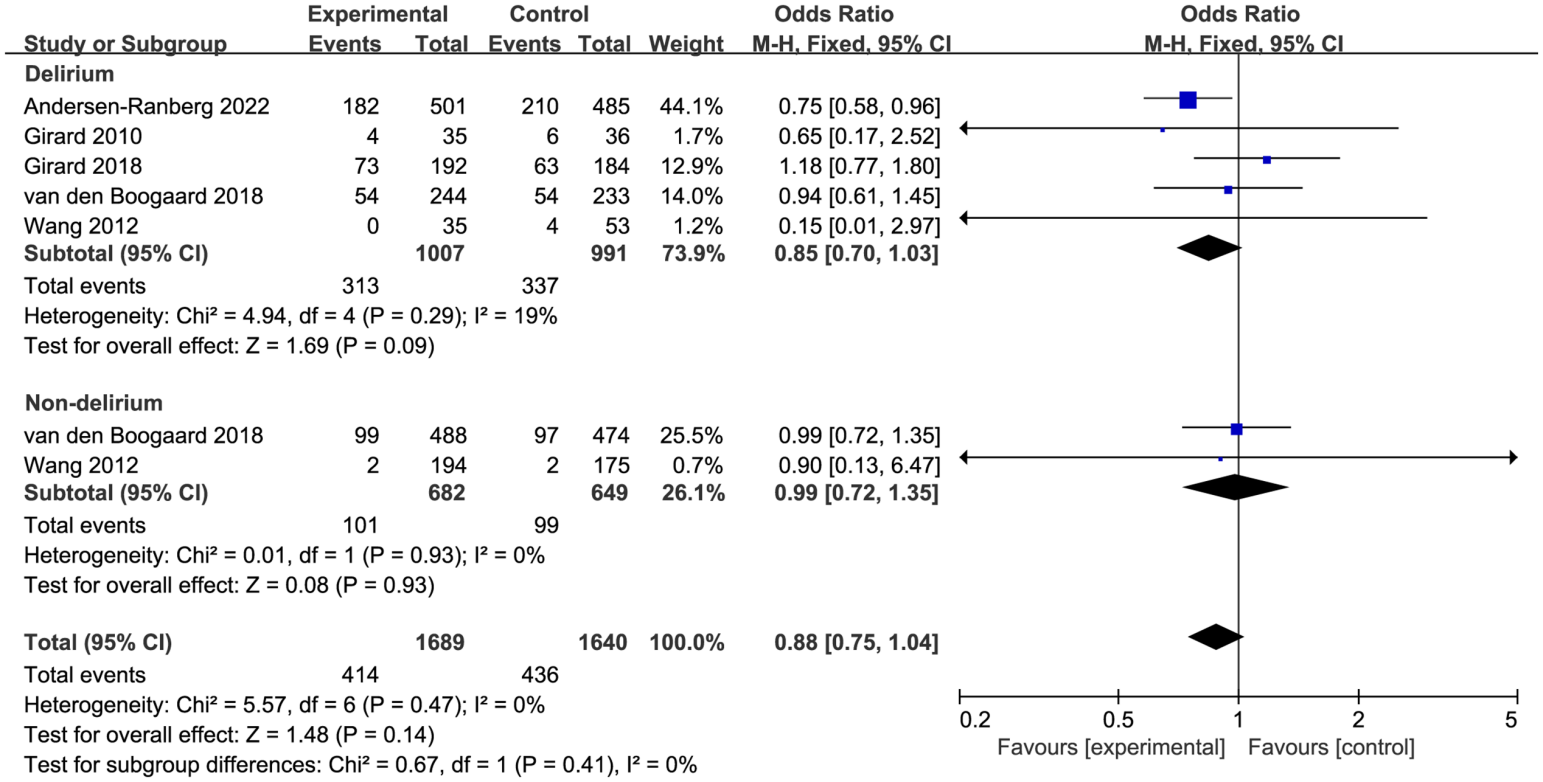
Forest plot showing the subgroup analysis of overall mortality, patients with delirium versus patients without delirium.

Prespecified subgroup analysis stratified by population (patients with delirium or without delirium) was performed to investigate the potential source of heterogeneity (Table 2). Compared with placebo, a trend toward reduced overall mortality by haloperidol was observed in patients with delirium (OR 0.85, 95%CI 0.70 to 1.03, *p* = 0.09, *I*^2^ = 19%; Figure 4), although it was not statistically significant. Furthermore, the sensitivity analysis showed no significant difference in the short-term outcomes, indicating the good robustness (Supplementary material 4).

### Secondary outcomes

**Figure 5:**
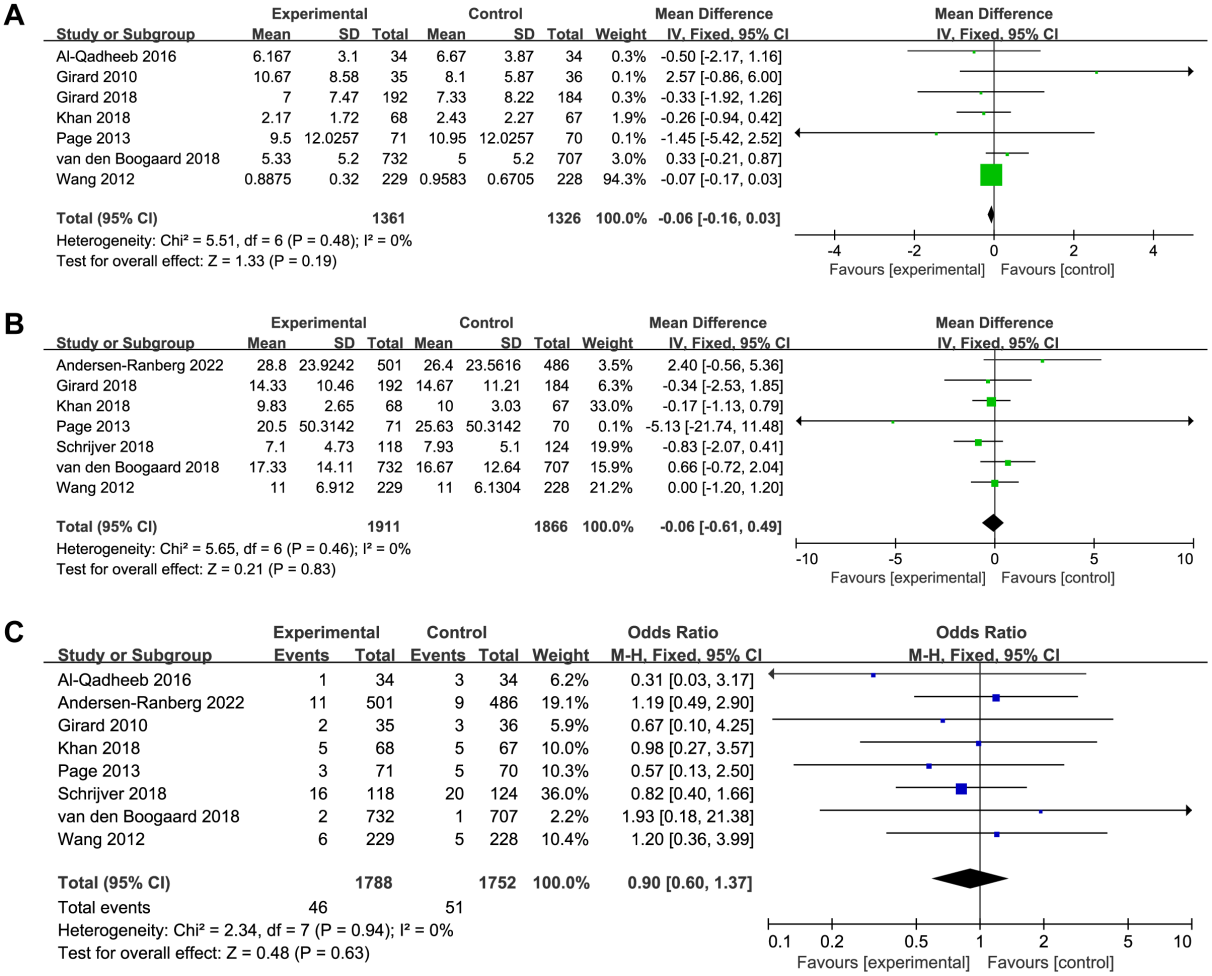
Forest plot showing the association between haloperidol and **(A)** length of ICU stay, **(B)** length of hospital stay, and **(C)** adverse events.

A total of seven trials reported the length of ICU and hospital stay, there was no significant difference between patients received haloperidol and placebo (ICU: MD −0.06, 95%CI −0.16 to 0.03, *p* = 0.19, *I*^2^ = 0%, Figure 5A; hospital: MD −0.06, 95%CI −0.61 to 0.49, *p* = 0.83, *I*^2^ = 0%, Figure 5B). There were eight trials reported the incidence of adverse events, the result indicated that the use of haloperidol did not increase the incidence of adverse events (OR 0.90, 95%CI 0.60 to 1.37, *p* = 0.63, *I*^2^ = 0%, Figure 5C). Upon the results of sensitivity analysis, we found that the results were consistent with those of the overall analysis, suggesting that our findings are robust (Supplementary material 4).

## Discussion

In this updated meta-analysis of RCTs, which involved 3,916 critically ill patients, results showed that haloperidol treatment had no impact on delirium-free days, overall mortality, or length of ICU and hospital stay when compared to placebo. However, it seemed to have a potential beneficial effect on overall mortality among critically ill patients with delirium, whereas it was not statistically significant. In addition, we found that usage of haloperidol did not increase the incidence of adverse events.

Among critically ill patients, delirium is a common occurrence and it is often addressed through the administration of pharmacological interventions, with haloperidol being the most commonly used pharmacologic intervention (15). Prior to our study, several systematic reviews and meta-analyses (32–36) have evaluated the efficacy of haloperidol in preventing and/or treating delirium in adult patients. However, these studies included in different population (surgical patients, patients in ward or ICU), and management (prevention and treatment). Our study distinguishes itself from previous literature by conducting a meta-analysis of high-quality randomized controlled trials (RCTs) that focused on a single management approach (treatment with haloperidol) and a specific population (critically ill adult patients) in the context of hospital-associated delirium. We excluded several trials carried out in the setting of elective surgery because the participants were not critically ill patients, and the effect of haloperidol in hospitalized non-ICU patients has been well assessed (37). Furthermore, in consideration of the potential clinical heterogeneity, we only included trials comparing haloperidol with placebo. The objective of our meta-analysis was to provide an updated and comprehensive analysis of the available RCTs on the safety and effectiveness of haloperidol for the treatment of delirium in adult critically ill patients.

Delirium is a common condition among critically ill patients that is associated with increased morbidity and mortality. Despite numerous hypotheses, the pathogenesis of delirium remains unknown (38, 39). It is believed that alterations in neurotransmitters, specifically an excess of dopamine and cholinergic deficiency, play a central role. Haloperidol, a D2 dopamine receptor antagonist, is a potential pharmacological option for the prevention and treatment of ICU-related delirium due to its ability to disinhibit acetylcholine and reduce the use of psychotropic sedatives/analgesics (40, 41). However, there is currently no clear evidence to support the use of haloperidol for this indication, and guidelines from the Society of Critical Care discourage its use in critically ill patients. Likewise, our findings do not support the notion that haloperidol provides either beneficial or adverse effects, and the level of uncertainty surrounding its clinical utility remains considerable. Although the results of subgroup analysis indicated that the use of haloperidol might have a potential beneficial effect on overall mortality among critically ill patients with delirium, whereas it was not statistically significant.

The lack of evidence concerning the utilization of haloperidol as a therapeutic option for delirium poses a considerable challenge to clinicians responsible for managing critically ill patients. To effectively manage delirium in critically ill patients, clinicians are advised to prioritize non-pharmacological interventions and strategies, such as early mobilization and mild sedation (10, 11). Furthermore, clinicians are encouraged to optimize modifiable risk factors and exercise caution when considering the use of antipsychotic medications in the management of delirium (42, 43). Although the low level of certainty surrounding the efficacy of haloperidol, it is essential to implement systematic screening protocols to detect patients exhibiting signs of delirium. Moreover, haloperidol may still be considered as a viable treatment option in instances where preventative measures and non-pharmacological interventions have been exhausted, in line with current recommendations (1).

### Strengths and limitations

Our study has some strengths, including a broad and comprehensive strategy for study selection, exhaustive inclusion criteria, and high-quality statistical analysis methodology. Notably, our study stands out from previous research by incorporating the most up-to-date randomized trials, including the AID-ICU trial. This large-scale trial, which involved almost 1,000 patients from various ICUs, features significant improvements in methodology, including strict allocation concealment and blinding methods for the trial drug and better separation between groups for antipsychotic exposure. Our study provides the latest evidence on antipsychotic therapy in intensive care patients and highlights the importance of rigorous methodology in clinical trials. Moreover, considering the clinical heterogeneity in different types of patients could have affected the results, we performed subgroup analyses stratified by population and provided the evidence of potential benefit with haloperidol in patients with delirium. Such findings provide important practical recommendations for clinical management for patients with delirium.

However, the current study had certain limitations as well. First, four of the included trials (22, 24–26) are typically defined as small studies (<100 patients per arm), which may lead to small study effect bias (44). The pooled results of studies with small sample size might underestimate the beneficial effect of haloperidol in reducing mortality (Supplementary material 4). Secondly, the doses, timing and route of administration of haloperidol varied among including trials, as well as the types of patients and severity of illness. Moreover, the studies included in our analysis had varying criteria for intervention discontinuation or resumption, which may have introduced heterogeneity in the results.

Another limitation of our study is the lack of patient-level data, which prevented us from assessing the impact of sedative use on clinical outcomes. Notably, the use of certain sedatives, such as dexmedetomidine and light sedation, may have contributed to delirium prevention, whereas benzodiazepines may have lowered the delirium threshold. It should be noted that several trials included in our analysis utilized open-label antipsychotics and non-pharmacological interventions, which may have confounded our outcomes and should be controlled in future research. Moreover, some studies reported continuous variables using median and interquartile range, which were converted to mean and standard deviation, potentially introducing bias into our results. Finally, since most of the included studies used the CAM-ICU and ICDSC for detecting delirium, which was more sensitive in detecting active or hyperactive delirium (45). The hypoactive delirium has a high potential to be under-recognized and undiagnosed. The results of our meta-analysis are more applicable to the critically ill patients with active or hyperactive delirium, instead of hypoactive delirium.

## Conclusion

In conclusion, the use of haloperidol compared to placebo did not significantly increase the delirium-free days, reduce the overall mortality, shorten length of ICU stay and length of hospital stay in critically ill patients. Additionally, there was no increased risk of adverse events. Individualized clinical decision-making is critical in the administration of haloperidol for delirium treatment, considering the patient’s condition, delirium subtype, and potential adverse effects.

In our opinion, further large-scale, well-designed RCTs are warranted to provide a more comprehensive understanding of the efficacy and safety of haloperidol for the prevention and treatment of different subtype of delirium in critically ill patients.

## Data availability statement

The original contributions presented in the study are included in the article/Supplementary material, further inquiries can be directed to the corresponding author.

## Author contributions

JH conceived the idea, performed the analysis, and drafted the initial draft writing of this paper. HZ and XZ contributed to the collection and interpretation of data. KZ provided technical support and helped to draft the work. XP contributed to the revision of this paper and the final approval of the version to be published. All authors contributed to the article and approved the submitted version.

## Funding

This work was supported in part by grants from the Hangzhou bio-medicine and health industry development support science and technology project (2022WJC076).

## Conflict of interest

The authors declare that the research was conducted in the absence of any commercial or financial relationships that could be construed as a potential conflict of interest.

## Publisher’s note

All claims expressed in this article are solely those of the authors and do not necessarily represent those of their affiliated organizations, or those of the publisher, the editors and the reviewers. Any product that may be evaluated in this article, or claim that may be made by its manufacturer, is not guaranteed or endorsed by the publisher.

## Footnotes

^1^ https://osf.io/jwk65
