# Supplementary material for: The efficacy and safety of haloperidol for the treatment of delirium in critically ill patients: a systematic review and meta-analysis of randomized controlled trials
Source: Front Med (Lausanne). 2023 Jul 27;10:1200314. doi: 10.3389/fmed.2023.1200314 (PMC10414537; doi:10.3389/fmed.2023.1200314)
Supplement: Supplementary file 2 [file Data_Sheet_2.docx]

**Supplementary Material 2: Searching strategies**

**Pubmed 86**

#1 Intensive Care Units [MeSH Terms] OR Critical Care [MeSH Terms] OR Critical Illness [MeSH Terms] OR icu [Title/Abstract] OR Critical Care [Title/Abstract] OR critically ill [Title/Abstract] OR Intensive Care [Title/Abstract]

#2 Haloperidol [Title/Abstract] OR Haloperidol [MeSH Terms]

#3 Delirium [MeSH Terms] OR Delirium [Title/Abstract]

#4 randomized controlled trial [MeSH Terms] OR random* [Title/Abstract]

#1 AND #2 AND #3 AND #4

**Embase 96**

#1 ‘Intensive Care Units’:ti,ab,kw OR ‘Critical Care’:ti,ab,kw OR ' intensive care unit '/exp OR ' Intensive Care '/exp OR ‘Critical Illness’:ti,ab,kw OR ‘critically ill’:ti,ab,kw OR ‘Intensive Care’:ti,ab,kw OR ‘ICU’:ti,ab,kw

#2 ' Haloperidol ':ti,ab,kw OR ' Haloperidol '/exp

#3 ' Delirium ':ti,ab,kw OR ' Delirium '/exp

#4 'randomized controlled trial'/de OR 'randomized controlled trial'/exp

#1 AND #2 AND #3 AND #4

**Scopus 119**

#1 TITLE-ABS-KEY (Intensive Care Units) OR TITLE-ABS-KEY (Critical Care) OR TITLE-ABS-KEY (Critical Illness) OR TITLE-ABS-KEY (icu) OR TITLE-ABS-KEY (critically ill) OR TITLE-ABS-KEY (Intensive Care)

#2 TITLE-ABS-KEY (Haloperidol)

#3 TITLE-ABS-KEY (Delirium)

#4 TITLE-ABS-KEY (randomized) OR TITLE-ABS-KEY (random) OR TITLE-ABS-KEY (randomised)

#1 AND #2 AND #3 AND #4

**Cochrane Library 120**

#1 (Intensive Care Units):ti,ab,kw OR (Critical Care):ti,ab,kw OR (Critical Illness):ti,ab,kw OR (icu):ti,ab,kw OR (critically ill):ti,ab,kw OR (Intensive Care):ti,ab,kw

#2 (Haloperidol):ti,ab,kw

#3 (Delirium):ti,ab,kw

#4 (randomized):ti,ab,kw OR (randomised):ti,ab,kw OR (random):ti,ab,kw

#1 AND #2 AND #3 AND #4
