# Supplementary material for: The efficacy and safety of haloperidol for the treatment of delirium in critically ill patients: a systematic review and meta-analysis of randomized controlled trials
Source: Front Med (Lausanne). 2023 Jul 27;10:1200314. doi: 10.3389/fmed.2023.1200314 (PMC10414537; doi:10.3389/fmed.2023.1200314)
Supplement: Supplementary file 3 [file Data_Sheet_3.docx]

**Supplementary Material 3:** List of excluded studies with reasons

| **Author, year and reference** | **Reason for exclusion** |
| --- | --- |
| Hollinger et al. (2021)[1] | Participants were not critically ill patients (adult patients that underwent elective or emergency surgery under general or combined anesthesia) |
| Fukata et al. (2017)[2] | Participants were not critically ill patients (patients of ≥75 years of age who underwent elective abdominal surgery under  general anesthesia or elective orthopedic surgery under general/spinal anesthesia) |
| Fukata et al. (2014)[3] | Participants were not critically ill patients (patients aged 75 years or older who underwent elective surgery for digestive or orthopedic disease) |
| Kalisvaart et al. (2005)[4] | Participants were not critically ill patients (adult patients aged 70 and older admitted for acute or elective hip surgery) |
| Ortiz et al. (2022)[5] | No concerned outcomes or relevant data not reported (no concerned outcomes for intervention and control groups) |
| Duprey et al. (2020)[6] | No concerned outcomes or relevant data not reported (secondary cohort analysis of an included study, no concerned outcomes) |
| Rood et al. (2019)[7] | No concerned outcomes or relevant data not reported (secondary analysis of an included study, no concerned outcomes) |
| Smit et al. (2021)[8] | Only abstract |
| Collet et al. (2017)[9] | Only abstract |
| Klein et al. (2016)[10] | Only abstract |
| Duprey et al. (2016)[11] | Only abstract |
| Gibson et al. (2018)[12] | Only abstract |
| Sadlonova et al. (2022)[13] | Review, meta-analysis, or protocol (review) |
| Smit et al. (2021)[14] | Review, meta-analysis, or protocol (protocol) |
| Kim et al. (2020)[15] | Review, meta-analysis, or protocol (meta-analysis) |
| Chen et al. (2020)[16] | Review, meta-analysis, or protocol (meta-analysis) |
| Lin et al. (2020)[17] | Review, meta-analysis, or protocol (meta-analysis) |
| Zayed et al. (2019)[18] | Review, meta-analysis, or protocol (meta-analysis) |
| Burry et al. (2018)[19] | Review, meta-analysis, or protocol (review) |
| Schrijver et al. (2016)[20] | Review, meta-analysis, or protocol (review) |
| Schrijver et al. (2014)[21] | Review, meta-analysis, or protocol (protocol) |
| Bledowski et al. (2012)[22] | Review, meta-analysis, or protocol (review) |
| van den Boogaard et al. (2013)[23] | Improper intervention and control methods (before/after evaluation) |
| Soltani et al. (2021)[24] | Improper intervention and control methods (control drug was dexmedetomidine) |
| Devlin et al. (2010)[25] | Improper intervention and control methods (control drug was quetiapine) |
| Atalan at al. (2013)[26] | Improper intervention and control methods (control drug was morphine) |
| Tagarakis et al. (2012)[27] | Improper intervention and control methods (control drug was ondasetron) |
| Reade et al. (2009)[28] | Improper intervention and control methods (control drug was dexmedetomidine) |
| Skrobik et al. (2004)[29] | Improper intervention and control methods (control drug was olanzapine) |

**Reference**

1. Hollinger A, Rüst CA, Riegger H, Gysi B, Tran F, Brügger J, Huber J, Toft K, Surbeck M, Schmid HR *et al*: **Ketamine vs. haloperidol for prevention of cognitive dysfunction and postoperative delirium: A phase IV multicentre randomised placebo-controlled double-blind clinical trial**. *J Clin Anesth* 2021, **68**:110099.

2. Fukata S, Kawabata Y, Fujishiro K, Kitagawa Y, Kuroiwa K, Akiyama H, Takemura M, Ando M, Hattori H: **Haloperidol prophylaxis for preventing aggravation of postoperative delirium in elderly patients: a randomized, open-label prospective trial**. *Surg Today* 2017, **47**(7):815-826.

3. Fukata S, Kawabata Y, Fujisiro K, Katagawa Y, Kuroiwa K, Akiyama H, Terabe Y, Ando M, Kawamura T, Hattori H: **Haloperidol prophylaxis does not prevent postoperative delirium in elderly patients: a randomized, open-label prospective trial**. *Surg Today* 2014, **44**(12):2305-2313.

4. Kalisvaart KJ, de Jonghe JF, Bogaards MJ, Vreeswijk R, Egberts TC, Burger BJ, Eikelenboom P, van Gool WA: **Haloperidol prophylaxis for elderly hip-surgery patients at risk for delirium: a randomized placebo-controlled study**. *J Am Geriatr Soc* 2005, **53**(10):1658-1666.

5. Ortiz D, Lindroth HL, Braly T, Perkins AJ, Mohanty S, Meagher AD, Khan SH, Boustani MA, Khan BA: **Delirium severity does not differ between medical and surgical intensive care units after adjusting for medication use**. *Sci Rep* 2022, **12**(1):14447.

6. Duprey MS, van den Boogaard M, van der Hoeven JG, Pickkers P, Briesacher BA, Saczynski JS, Griffith JL, Devlin JW: **Association between incident delirium and 28- and 90-day mortality in critically ill adults: a secondary analysis**. *Crit Care* 2020, **24**(1):161.

7. Rood PJT, Zegers M, Slooter AJC, Beishuizen A, Simons KS, van der Voort PHJ, van der Woude MCE, Spronk PE, van der Hoeven JG, Pickkers P *et al*: **Prophylactic Haloperidol Effects on Long-term Quality of Life in Critically Ill Patients at High Risk for Delirium: Results of the REDUCE Study**. *Anesthesiology* 2019, **131**(2):328-335.

8. Smit L, Slooter A, Devlin J, Trogrlic Z, Hunfeld N, Osse RJ, Ponssen H, Brouwers A, Schoonderbeek J, Simons K *et al*: **Efficacy of halopeRIdol to decrease the burden of Delirium in adult Critically ill patiEnts (EuRIDICE): A Randomized Clinical Trial**. *Intensive Care Medicine Experimental* 2021, **9**(SUPPL 1).

9. Collet MO, Wøien H, Schandl A, Hästbacka J, Van Den Boogaard M, Hänggi M, Nydahl P, Citerio G, Sonneville R, Bozza FA *et al*: **Pharmacological interventions for delirium in intensive care unit (AID-ICU): An international inception cohort study**. *Intensive Care Medicine Experimental* 2017, **5**(2).

10. Klein L, Driver B, Moore J, Parrill C, Fagerstrom E, Ho J, Miner J: **Characteristics of emergency department patients with agitation and excited delirium syndrome**. *Annals of Emergency Medicine* 2016, **68**(4):S93.

11. Duprey M, Al-Qadheeb N, Roberts R, Skrobik Y, Schumaker G, Devlin J: **QTC interval prolongation with low-dose iv haloperidol: Post hoc analysis of a placebo control trial**. *Critical Care Medicine* 2016, **44**(12):290.

12. Gibson W, Giarratano M, Glass M: **Evaluation of ramelteon for the reduction of ICU delirium**. *Critical Care Medicine* 2018, **46**:446.

13. Sadlonova M, Duque L, Smith D, Madva EN, Amonoo HL, Vogelsang J, Staton SC, von Arnim CAF, Huffman JC, Celano CM: **Pharmacologic treatment of delirium symptoms: A systematic review**. *Gen Hosp Psychiatry* 2022, **79**:60-75.

14. Smit L, Trogrlić Z, Devlin JW, Osse RJ, Ponssen HH, Slooter AJC, Hunfeld NGM, Rietdijk WJR, Gommers D, van der Jagt M: **Efficacy of halopeRIdol to decrease the burden of Delirium In adult Critically ill patiEnts (EuRIDICE): study protocol for a prospective randomised multi-centre double-blind placebo-controlled clinical trial in the Netherlands**. *BMJ Open* 2020, **10**(9):e036735.

15. Kim MS, Rhim HC, Park A, Kim H, Han KM, Patkar AA, Pae CU, Han C: **Comparative efficacy and acceptability of pharmacological interventions for the treatment and prevention of delirium: A systematic review and network meta-analysis**. *J Psychiatr Res* 2020, **125**:164-176.

16. Chen Z, Chen R, Zheng D, Su Y, Wen S, Guo H, Ye Z, Deng Y, Liu G, Zuo L *et al*: **Efficacy and safety of haloperidol for delirium prevention in adult patients: An updated meta-analysis with trial sequential analysis of randomized controlled trials**. *J Clin Anesth* 2020, **61**:109623.

17. Lin P, Zhang J, Shi F, Liang ZA: **Can haloperidol prophylaxis reduce the incidence of delirium in critically ill patients in intensive care units? A systematic review and meta-analysis**. *Heart Lung* 2020, **49**(3):265-272.

18. Zayed Y, Barbarawi M, Kheiri B, Banifadel M, Haykal T, Chahine A, Rashdan L, Aburahma A, Bachuwa G, Seedahmed E: **Haloperidol for the management of delirium in adult intensive care unit patients: A systematic review and meta-analysis of randomized controlled trials**. *J Crit Care* 2019, **50**:280-286.

19. Burry L, Mehta S, Perreault MM, Luxenberg JS, Siddiqi N, Hutton B, Fergusson DA, Bell C, Rose L: **Antipsychotics for treatment of delirium in hospitalised non-ICU patients**. *Cochrane Database Syst Rev* 2018, **6**(6):Cd005594.

20. Schrijver EJ, de Graaf K, de Vries OJ, Maier AB, Nanayakkara PW: **Efficacy and safety of haloperidol for in-hospital delirium prevention and treatment: A systematic review of current evidence**. *Eur J Intern Med* 2016, **27**:14-23.

21. Schrijver EJ, de Vries OJ, Verburg A, de Graaf K, Bet PM, van de Ven PM, Kamper AM, Diepeveen SH, Anten S, Siegel A *et al*: **Efficacy and safety of haloperidol prophylaxis for delirium prevention in older medical and surgical at-risk patients acutely admitted to hospital through the emergency department: study protocol of a multicenter, randomised, double-blind, placebo-controlled clinical trial**. *BMC Geriatr* 2014, **14**:96.

22. Bledowski J, Trutia A: **A review of pharmacologic management and prevention strategies for delirium in the intensive care unit**. *Psychosomatics* 2012, **53**(3):203-211.

23. van den Boogaard M, Schoonhoven L, van Achterberg T, van der Hoeven JG, Pickkers P: **Haloperidol prophylaxis in critically ill patients with a high risk for delirium**. *Crit Care* 2013, **17**(1):R9.

24. Soltani F, Tabatabaei S, Jannatmakan F, Nasajian N, Amiri F, Darkhor R, Moravej M: **Comparison of the Effects of Haloperidol and Dexmedetomidine on Delirium and Agitation in Patients with a Traumatic Brain Injury Admitted to the Intensive Care Unit**. *Anesth Pain Med* 2021, **11**(3):e113802.

25. Devlin JW, Roberts RJ, Fong JJ, Skrobik Y, Riker RR, Hill NS, Robbins T, Garpestad E: **Efficacy and safety of quetiapine in critically ill patients with delirium: a prospective, multicenter, randomized, double-blind, placebo-controlled pilot study**. *Crit Care Med* 2010, **38**(2):419-427.

26. Atalan N, Efe Sevim M, Akgün S, Fazlıoğulları O, Başaran C: **Morphine is a reasonable alternative to haloperidol in the treatment of postoperative hyperactive-type delirium after cardiac surgery**. *J Cardiothorac Vasc Anesth* 2013, **27**(5):933-938.

27. Tagarakis GI, Voucharas C, Tsolaki F, Daskalopoulos ME, Papaliagkas V, Parisis C, Gogaki E, Tsagalas I, Sataitidis I, Tsolaki M *et al*: **Ondasetron versus haloperidol for the treatment of postcardiotomy delirium: a prospective, randomized, double-blinded study**. *J Cardiothorac Surg* 2012, **7**:25.

28. Reade MC, O'Sullivan K, Bates S, Goldsmith D, Ainslie WR, Bellomo R: **Dexmedetomidine vs. haloperidol in delirious, agitated, intubated patients: a randomised open-label trial**. *Crit Care* 2009, **13**(3):R75.

29. Skrobik YK, Bergeron N, Dumont M, Gottfried SB: **Olanzapine vs haloperidol: treating delirium in a critical care setting**. *Intensive Care Med* 2004, **30**(3):444-449.
