# Supplementary material for: The efficacy and safety of haloperidol for the treatment of delirium in critically ill patients: a systematic review and meta-analysis of randomized controlled trials
Source: Front Med (Lausanne). 2023 Jul 27;10:1200314. doi: 10.3389/fmed.2023.1200314 (PMC10414537; doi:10.3389/fmed.2023.1200314)
Supplement: Supplementary file 4 [file Data_Sheet_4.docx]

**Supplementary Material 4:** Publication bias assessment by funnel plot and Egger’s test, sensitivity analyses, subgroup analyses.


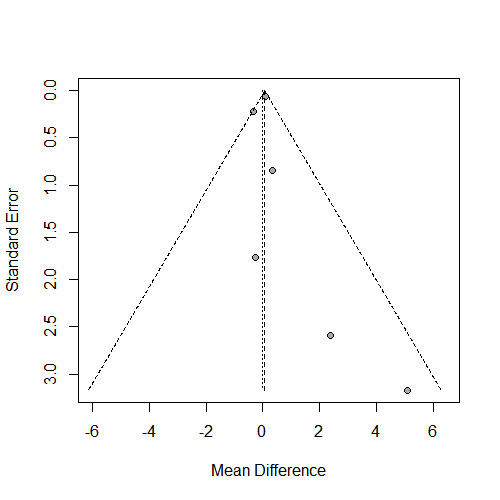


Figure 1: Funnel plot for delirium-free days, Egger’s test P=0.6703


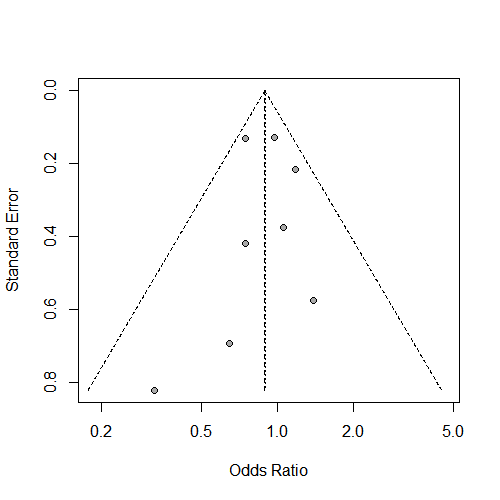


Figure 2: Funnel plot for overall mortality, Egger’s test P=0.8529


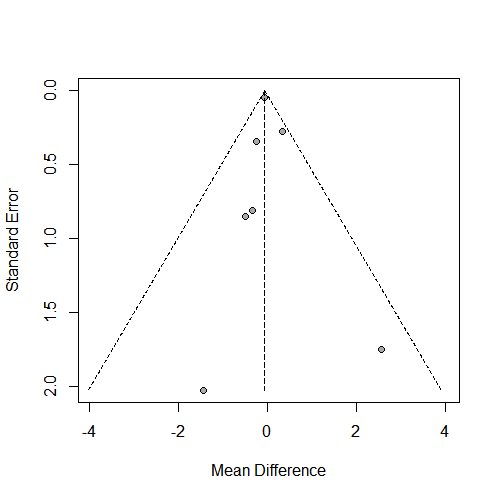


Figure 3: Funnel plot for length of ICU stay, Egger’s test P= 0.7768


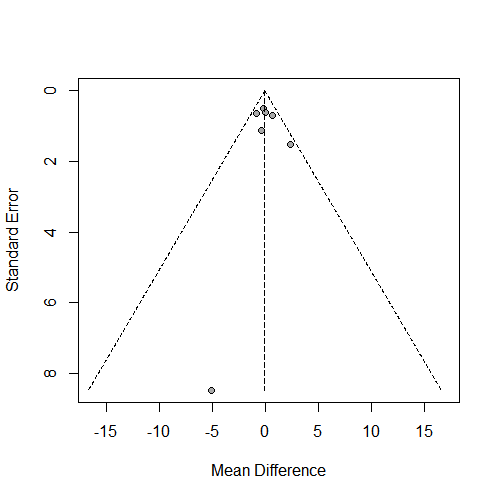


Figure 4: Funnel plot for length of hospital stay, Egger’s test P=0.7367


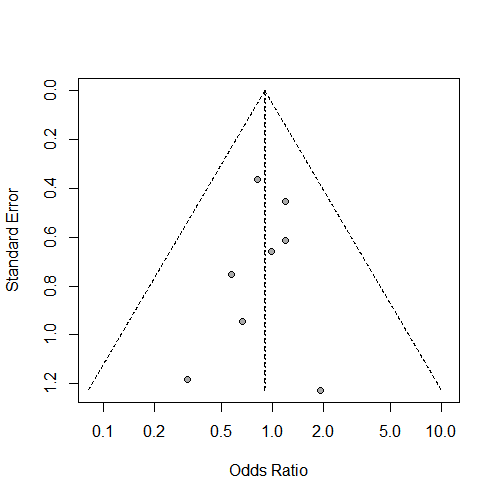


Figure 5: Funnel plot for adverse events, Egger’s test P=0.6172


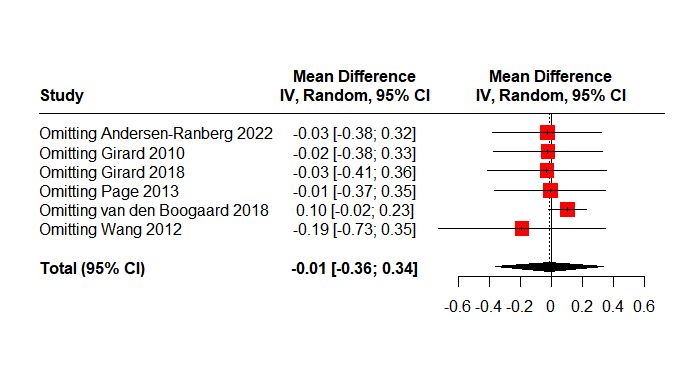


Figure 6: Sensitivity analysis for delirium-free days


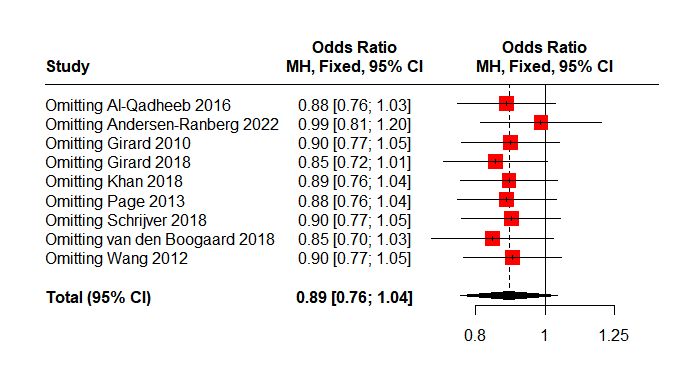


Figure 7: Sensitivity analysis for overall mortality


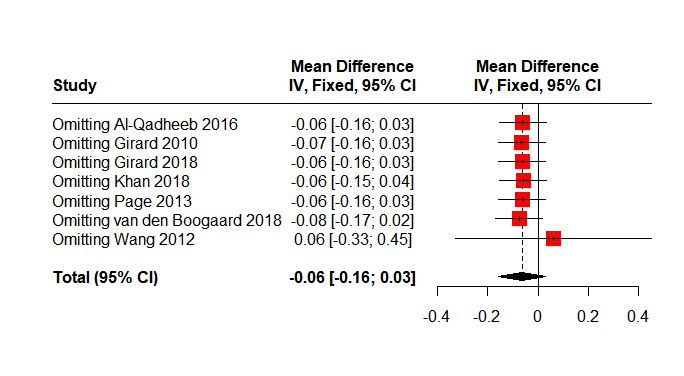


Figure 8: Sensitivity analysis for length of ICU stay


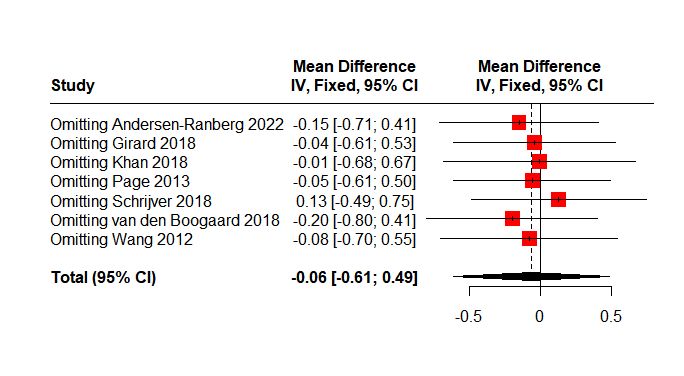


Figure 9: Sensitivity analysis for length of hospital stay


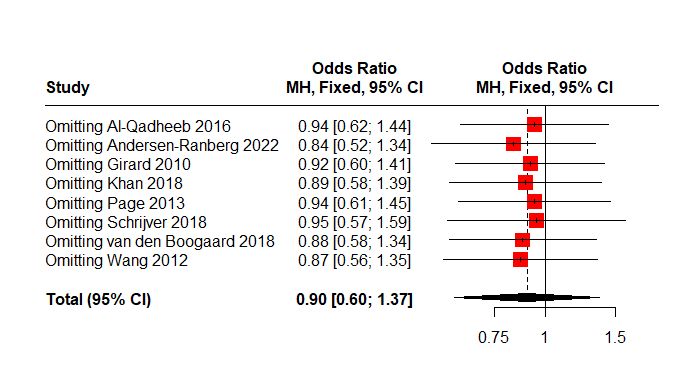


Figure 10: Sensitivity analysis for adverse events


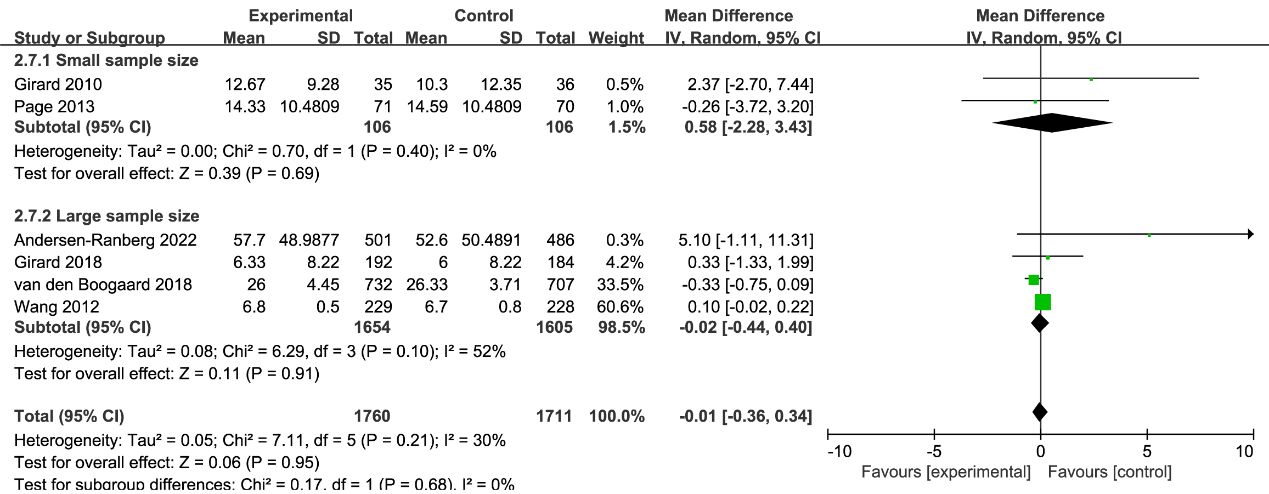


Figure 10: Subgroup analysis for delirium-free days, small sample size studies versus large sample size studies


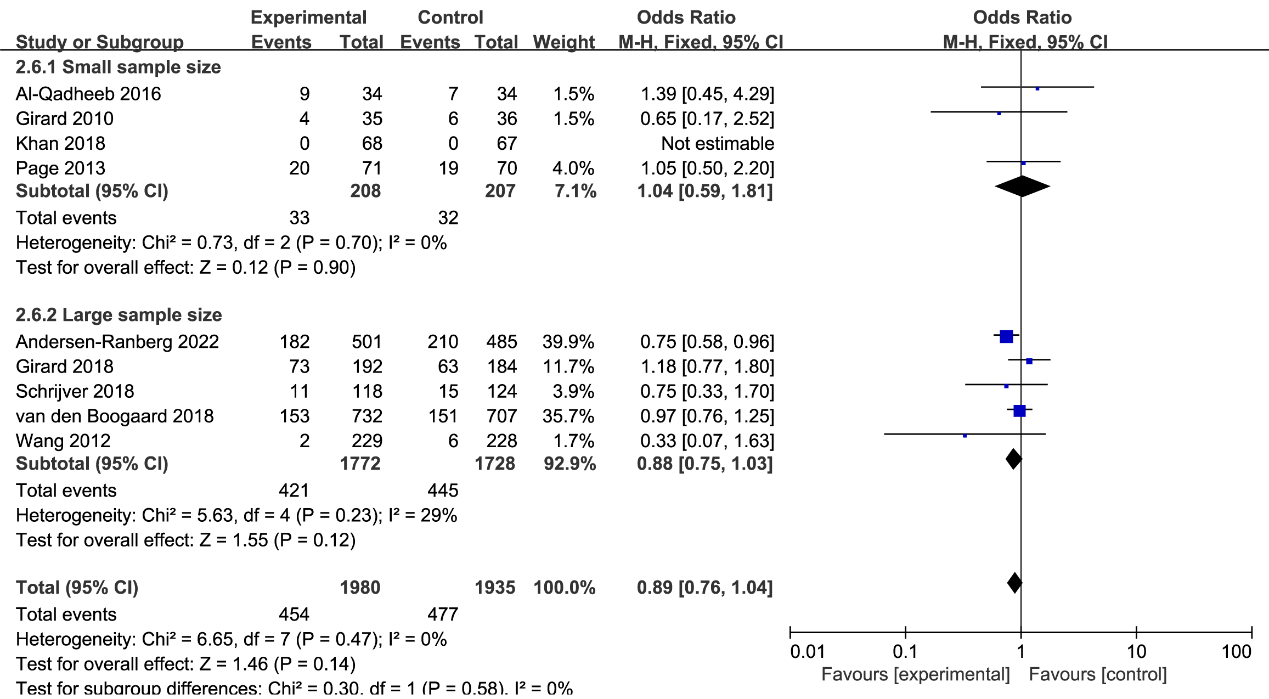


Figure 11: Subgroup analysis for overall mortality, small sample size studies versus large sample size studies


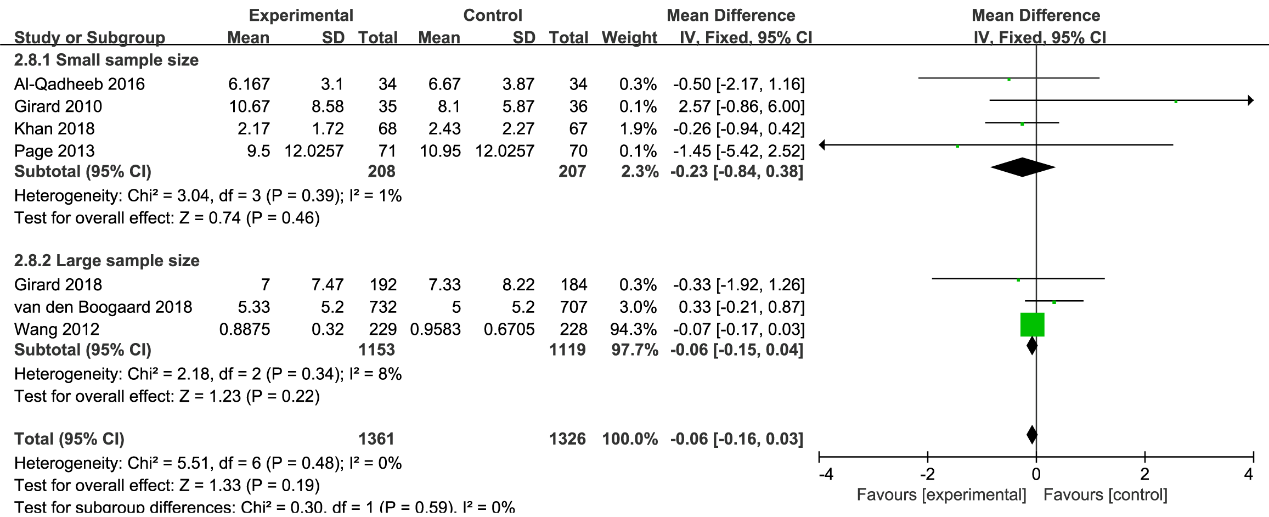


Figure 12: Subgroup analysis for length of ICU stay, small sample size studies versus large sample size studies


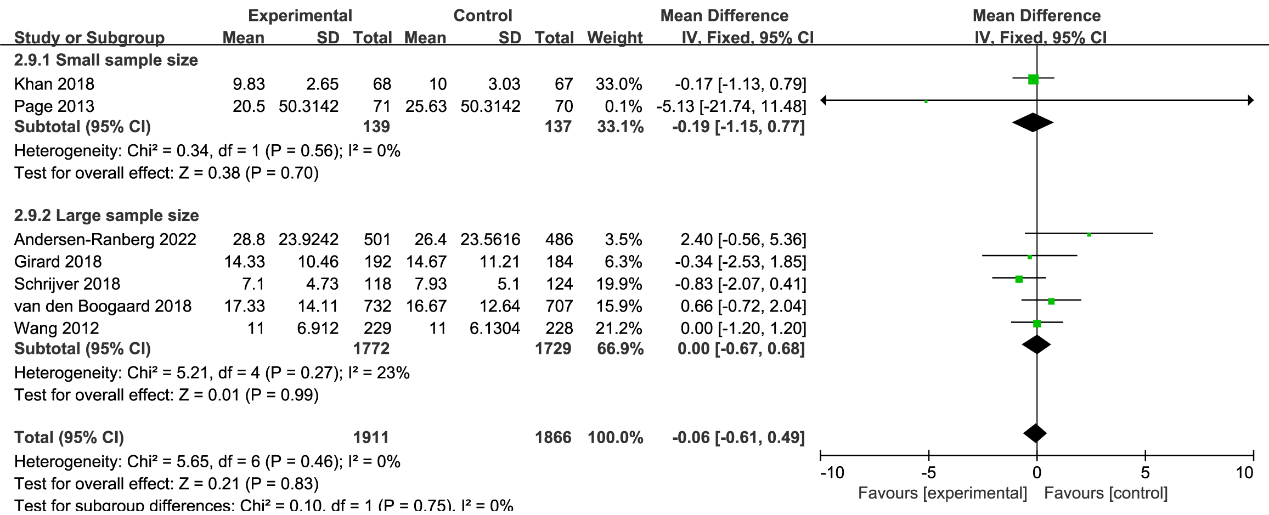


Figure 13: Subgroup analysis for length of hospital stay, small sample size studies versus large sample size studies


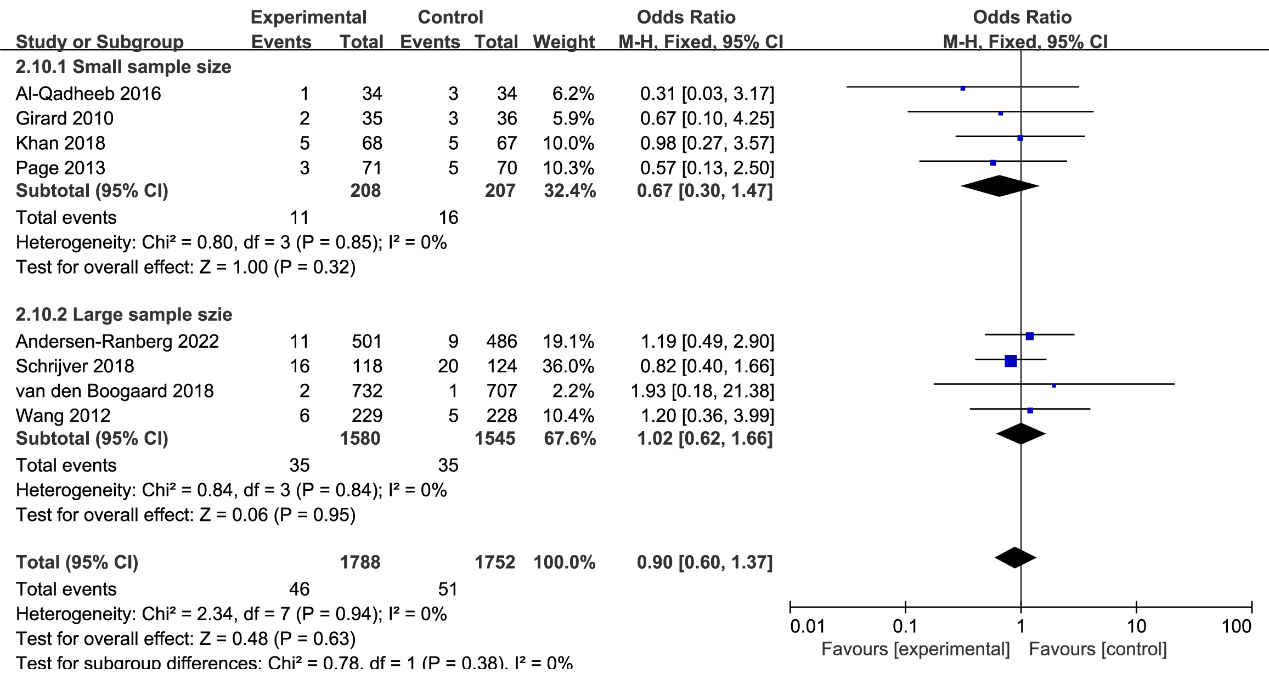


Figure 14: Subgroup analysis for adverse events, small sample size studies versus large sample size studies
